# A retrospective cohort analysis of ionised calcium levels in major trauma patients who have received early blood product transfusion in the Emergency Department

**DOI:** 10.1186/1757-7241-23-S2-O3

**Published:** 2015-09-11

**Authors:** Stacey J Webster, Samuel JH Todd, Chris R Wright

**Affiliations:** 1 Lt, Royal Army Medical Corps; Frimley Health NHS FT; 2 Flt Lt, Royal Air Force Medical Branch; Imperial Healthcare NHS Trust; 3 Lt Col, Royal Army Medical Corps; Imperial Healthcare NHS Trust

## Background

Exsanguination and coagulopathy remain one of the leading causes of preventable trauma related death [1]. Low ionised calcium levels have been associated with hypotension and increased mortality[2]. Blood product contains citrate that acts as a calcium chelating agent. We hypothesized that trauma patients are at risk of hypocalcaemia and blood products given to resuscitate them would reduce serum Calcium concentration, and therefore affect 30-day mortality.

## Methods

A retrospective cohort analysis was performed on all major trauma patients who had received early blood product in the Emergency Department of a single London Major Trauma Centre over a one year period (January 2013 – January 2014). Ionised calcium levels were taken from venous blood gases from before and after blood product had been transfused. Excel was used to analyse the data.

## Results

The study included 60 patients aged between 10 and 92 (mean 40), 46 male (77%) and 14 female (23%). Mechanism of injury was predominantly blunt 48 (80%) and penetrating 12 (20%). Patients received between 1 and 16 units of blood product (mode 2). Mean ISS was 26 (5-50) and overall 30 day mortality was 12%.

60% were hypocalcaemic on arrival before receiving any blood product (Mean [Ca] 1.1mmol/L 95% CI 1.08 – 1.13) 89% of patients were hypocalcaemic after receiving blood product (Mean [Ca] 0.95mmol/L 95% CI 0.9 – 1.01). There was a statistically significant difference between ionized calcium levels pre and post blood transfusion. A drop in calcium was seen after receiving just one unit of packed red blood cells, with the average drop being 0.05 mmol/L per unit of blood product received.

## Conclusion

Trauma patients that have sustained blood loss are at risk of hypocalcaemia. Receiving just one unit of blood product further compounds their hypocalcaemic state and the more units that are given the greater the fall that is seen.

## References

[B1] Hess J R Brohi K Dutton R P Hauser C J The coagulopathy of trauma: a review of mechanisms Journal of Trauma- … 2008 10.1097/TA.0b013e3181877a9c 18849786

[B2] Magnotti L J Admission Ionized Calcium Levels Predict the Need for Multiple Transfusions: A Prospective Study of 591 Critically Ill Trauma Patients The Journal of Trauma: Injury, Infection, and Critical Care 2011 70 391 397 10.1097/TA.0b013e31820b5d98 21307739

